# Multi-Omics Analysis of Brain Metastasis Outcomes Following Craniotomy

**DOI:** 10.3389/fonc.2020.615472

**Published:** 2021-04-06

**Authors:** Jing Su, Qianqian Song, Shadi Qasem, Stacey O’Neill, Jingyun Lee, Cristina M. Furdui, Boris Pasche, Linda Metheny-Barlow, Adrianna H. Masters, Hui-Wen Lo, Fei Xing, Kounosuke Watabe, Lance D. Miller, Stephen B. Tatter, Adrian W. Laxton, Christopher T. Whitlow, Michael D. Chan, Michael H. Soike, Jimmy Ruiz

**Affiliations:** ^1^ Department of Cancer Biology, Wake Forest School of Medicine, Winston-Salem, NC, United States; ^2^ Department of Biostatistics, Indiana University School of Medicine, Indianapolis, IN, United States; ^3^ Department of Pathology, Wake Forest School of Medicine, Winston-Salem, NC, United States; ^4^ Proteomics and Metabolomics Shared Resource, Comprehensive Cancer Center, Wake Forest University School of Medicine, Winston-Salem, NC, United States; ^5^ Department of Internal Medicine, Section on Molecular Medicine, Wake Forest School of Medicine, Winston-Salem, NC, United States; ^6^ Department of Radiation Oncology, Wake Forest School of Medicine, Winston-Salem, NC, United States; ^7^ Department of Neurosurgery, Wake Forest School of Medicine, Winston-Salem, NC, United States; ^8^ Department of Radiology, Wake Forest School of Medicine, Winston-Salem, NC, United States; ^9^ Department of Radiation Oncology, University of Alabama-Birmingham, Birmingham, AL, United States; ^10^ Department of Medicine (Hematology & Oncology), Wake Forest School of Medicine, Winston-Salem, NC, United States; ^11^ Section of Hematology & Oncology, W.G. (Bill) Hefner Veterans Affair Medial Center (VAMC), Salisbury, NC, United States

**Keywords:** bioinformatics analysis, brain metastases, craniotomy, distant brain failure, RNA-Seq - RNA sequencing, proteomics, metabolomics, non-negative matrix factorization

## Abstract

**Background:**

The incidence of brain metastasis continues to increase as therapeutic strategies have improved for a number of solid tumors. The presence of brain metastasis is associated with worse prognosis but it is unclear if distinctive biomarkers can separate patients at risk for CNS related death.

**Methods:**

We executed a single institution retrospective collection of brain metastasis from patients who were diagnosed with lung, breast, and other primary tumors. The brain metastatic samples were sent for RNA sequencing, proteomic and metabolomic analysis of brain metastasis. The primary outcome was distant brain failure after definitive therapies that included craniotomy resection and radiation to surgical bed. Novel prognostic subtypes were discovered using transcriptomic data and sparse non-negative matrix factorization.

**Results:**

We discovered two molecular subtypes showing statistically significant differential prognosis irrespective of tumor subtype. The median survival time of the good and the poor prognostic subtypes were 7.89 and 42.27 months, respectively. Further integrated characterization and analysis of these two distinctive prognostic subtypes using transcriptomic, proteomic, and metabolomic molecular profiles of patients identified key pathways and metabolites. The analysis suggested that immune microenvironment landscape as well as proliferation and migration signaling pathways may be responsible to the observed survival difference.

**Conclusion:**

A multi-omics approach to characterization of brain metastasis provides an opportunity to identify clinically impactful biomarkers and associated prognostic subtypes and generate provocative integrative understanding of disease.

## Introduction

There are approximately 180,000 new cases of brain metastases in the US each year (1). However, patients with brain metastases represent a heterogeneous population and the prognosis may vary widely depending on primary tumor origin (2), molecular subset (3), histology (4), status of extracranial disease, number of lesions (5), brain metastasis volume (6), and the overall health status of the patient (7). Due to the heterogeneity in the presentation of these patients, several treatment options including surgery, stereotactic radiosurgery (SRS), and whole brain radiotherapy (WBRT) have been adopted.

The use of the various treatment options for brain metastases has evolved over time based on the relative strengths and toxicities related to each treatment regimen. Surgical resection is often reserved for larger or symptomatic brain metastases (1). SRS has proven most effective with fewer brain metastases, while WBRT is most often applied in patients with diffuse metastases (8). There is also the use of primary systemic targeted therapies for cancer that harbor actionable mutations like EGFR (9) or ALK (10). In spite of these general guidelines, a large proportion of patients will fall into a category in which their brain metastases enumerate into an intermediate category between few and many, and for these patients, little prospective data exists (11). Moreover, the variability in the outcomes of these patients makes it difficult to use the absolute number of brain metastases to guide management (12). There is a growing need to identify markers for disease response to systemic therapies, and there are many questions on how to use radiation and systemic therapies to treat patients and obtain optimal quality of life and survival endpoints.

There has been a number of studies that utilize single assay characterization of metastatic lesions (13, 14). This includes evaluation of brain metastasis at the gene level (15, 16). Few studies have integrated multiple levels of query to not only include the genome, but to evaluate the proteome and metabolome from the brain metastasis itself. Brain metastasis, largely from lung and breast cancers, poses a significant detrimental event as it relates to prognosis and quality of life. A limited number of studies have incorporated a proteomic or metabolomic based approach on human brain metastasis. There is a need to map out the signaling cascades of these tumors and if there is concordance regardless of tumor type.

Recent studies have suggested that in spite of the heterogeneity in the brain metastasis population, that there may be brain metastasis-specific mutations even across multiple histologies and primary cancer types (15, 16). Because brain metastasis patients as a population can segregate into various phenotypes of clinical behavior, the question has arisen as to whether the clinical behavior of brain metastases can be predicted. To address these questions, we profiled global proteomes, genomes, and the metabolome of resected brain metastases from a number of tumor types. We provided both individual and integrated analyses that revealed brain metastasis with similar RNA expression provided different post-transcriptional and post-translational levels.

## Methods

### Patient Population

The Wake Forest Brain Tumor Tissue bank was searched for samples between 2005 and 2016. This tumor bank included fresh frozen tissue, and included samples of patients who signed an informed consent to have a portion of their tumor tissue banked. Inclusion criteria for the study included brain metastasis samples from solid tumors in which clinical follow-up data were available. After craniotomy, patients with treated with post-operative radiotherapy (either cavity-directed SRS, WBRT) or placement of breast cancer patients treated with carmustine (BCNU)-containing wafers as previously described (17, 18). SRS was performed using the Leksell Gamma Knife B, C, or Perfexion units. Treatment planning was performed *via* the Leksell GammaPlan treatment planning system.

### Data Acquisition

This study was approved by the Wake Forest Institutional Review Board. Electronic medical records were used to determine patient clinical characteristics as well as clinical endpoints such as survival, local failure, distant brain failure, and the likelihood of neurologic death. In general, patients were imaged every three months for the first two years after craniotomy and then every 4–6 months thereafter. Distant brain failure was defined as the development of a new brain metastasis that was not present at the time of adjuvant therapy. Neurologic death was defined as per McTyre et al. (19).

### Proteomic Analysis

Prior to analysis, frozen tumor blocks were assessed by a board-certified pathologist (SQ) to assess for adequate and representative tissue. Approximately 20 mg of tissue was lysed in 1 ml of radioimmunoprecipitation assay (RIPA) buffer containing protease inhibitor using a bead mill homogenizer (Bead Ruptor, Omni International, Kennesaw, GA). RIPA lysate was then incubated sequentially with 10 mM dithiothreitol at 55°C for 30 min, and with 30 mM iodoacetamide at room temperature in the dark for another 30 min. A purified protein pellet was acquired from acetone precipitation. The pellet was subsequently treated with sequencing grade modified trypsin. The resultant peptides were de-salted using a C18 spin column, dried and then re-suspended in 5% (v/v) ACN containing 1% (v/v) formic acid for liquid chromatography-tandem mass spectroscopy (LC-MS/MS) analysis.

The LC-MS/MS analysis was performed utilizing a Q Exactive HF Hybrid Quadrupole-Orbitrap Mass Spectrometer (Thermo Scientific, Rockford, IL) interfaced with a Dionex Ultimate-3000 nano-UPLC system (Thermo Scientific, Rockford, IL) and a Nanospray Flex Ion Source (Thermo Scientific, Rockford, IL). An Acclaim PepMap 100 (C18, 5 μm, 100 Å, 100 μm x 2 cm) trap column and an Acclaim PepMap RSLC (C18, 2 μm, 100 Å, 75 μm x 15 cm) analytical column were used for the stationary phase. Chromatographic separation was achieved with a linear gradient consisting of mobile phases A (water with 0.1% formic acid) and B (acetonitrile with 0.1% formic acid) where the gradient was from 5% B at 0 min to 40% B at 80 min. MS/MS analysis was performed in data dependent mode for the twenty most intense ions from the full MS scan with dynamic exclusion option for 10 s enabled. Mass spectra were searched with the Sequest HT algorithm within the Proteome Discoverer v2.1 (Thermo Scientific), in combination with the human UniProt protein FASTA database (annotated 20,193 entries, December 2015).

### Genomic Analysis

Prior to analysis, frozen tumor block was assessed by a board-certified pathologist (SQ) to assess for adequate tumor content. Total RNA was purified from tumor specimens using the RNeasy Plus Micro Kit (Qiagen) with genomic DNA removal. RNA integrity (RIN) was determined by electrophoretic tracing using an Agilent Bioanalyzer. RNAseq libraries were constructed from samples (RIN >7.0) using the Illumina TruSeq Stranded Total RNA kit with Ribo-Zero rRNA depletion. Indexed libraries were sequenced on an Illumina NextSeq 500 DNA sequencer using 150x150-nt paired end reads, generating >40 million reads per sample (12 samples per flow cell) with >80% of sequences achieving >Q30 Phred quality scores. RNA-Seq analysis was performed following the standard pipeline (20) established by The Cancer Genome Atlas (TCGA) and the National Cancer Institute Genomic Data Commons (GDC) (21, 22). Briefly, quality of raw sequencing reads was assessed by FASTQC analysis (Babraham Bioinformatics). Sequence reads were aligned using the Spliced Transcripts Alignment to a Reference (STAR) sequence aligner (23) with a two-pass approach and gene counts determined using HTSeq software (24). The RNA-Seq data was reported as raw counts, FPKM (Fragments Per Kilobase of transcript per Million mapped reads), and FPKM-UQ (Upper 75% Quantile).

### Metabolomic Analysis

Prior to analysis, frozen tumor block was assessed by a board-certified pathologist (SQ) to assess for adequate tumor content. The metabolomic data were generated using the AbsoluteIDQ p180 Kit (Biocrates Life Sciences, Innsbruck, Austria).

### Patient Subtyping

The patient subtypes were determined by bi-clustering genes and samples using the RNA-Seq data and signed non-negative matrix factorization (sNMF)—an algorithm we previously developed (25, 26) to more reliably discern subpopulations defined by differential gene expression. The FPKM-UQ data, which was more robust than the FPKM one, were used for subtyping analysis. Low count inflation was controlled by discarding genes that were not detectable in more than half the samples. The FPKM-UQ expression data of each gene were then log-transformed, centered by its mean across all samples, and scaled by its root mean square. The normalized expression data were then bi-clustered using sNMF. The optimal subtype number were determined by screening the cluster number from 2 to 6 and evaluating the performance using seven different metrics [cophenetic coefficient (27), dispersion coefficient (28), explained variance (evar), residuals, residual sum of squares (RSS), silhouette, and sparseness (29)] with the randomized data as the negative control. The biclustering reached best performance when the subtype number was 4 (**Supplementary Figure 1**). The two largest subtypes were further analyzed.

### Survival Analysis

Kaplan-Meier estimator and log-rank test were used for the non-parametric survival analysis (R survival package version 2.44-1.1).

### Multi-Omics Differential Analysis

The proteomics and metabolomics data were used in enrichment analysis to identify differentially expressed proteins as well as metabolites showing differential abundance in the two largest subtypes. Briefly, low expressed proteins and low abundant metabolites which were not detectable in more than half samples were discarded. The data of each protein or metabolite were then log-transformed, centered by its mean across all samples, and scaled by its root mean square., Differential analysis between the two subtypes were performed using the R (version: 3.3.1) package “limma” (version: 3.30.13) (30), and significantly different proteins and metabolites were determined with false discovery rate (31) FDR <=0.05.

### Subtype Annotation

The expression data of mRNAs associated with the two largest subtypes as well as proteins differentially expressed in these two subtypes were used for enrichment analysis using Ingenuity Pathway Analysis (IPA) (32). Enriched canonical pathway was scored by its -log10(p-value). The overall score of a pathway was defined as the sum of the scores generated from the transcriptomic and the proteomics data, respectively.

## Results

### Patient Characteristics

**Figure 1:**
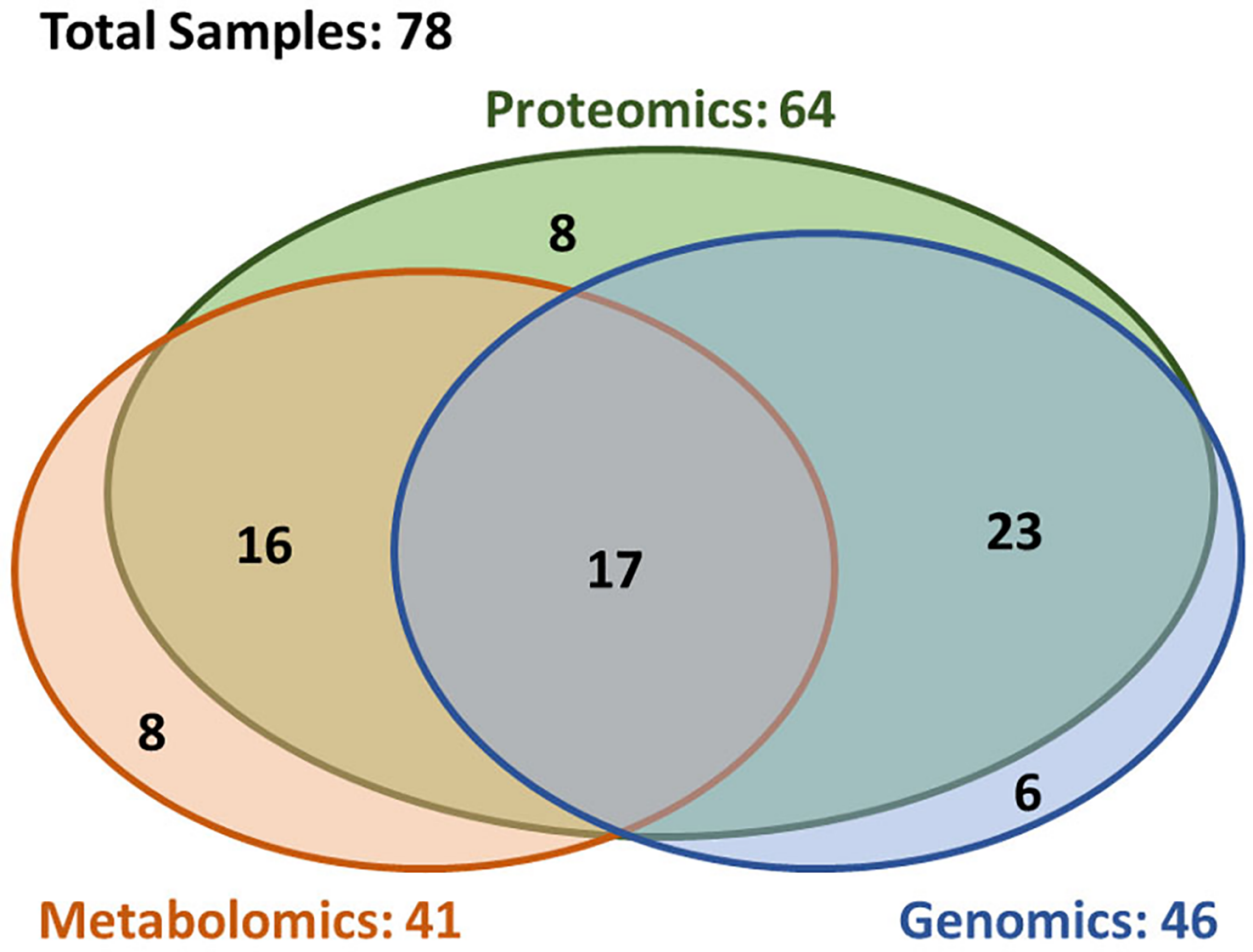
Overview of the availability of the multi-omics data. The multi-omics cohort (n = 78) was established through incorporating three closely correlated studies in genomics (n = 46), proteomics (n = 64), and metabolomics (n = 41). In this cohort, 72% patients (n = 56) had at least two types of omics data, and 22% patients (n = 17) had all three type of omics data.

A total of 78 patients were included in this analysis. **Figure 1** depicts a diagram of the patients in this study who had brain metastasis samples analyzed for either genomics, metabolomics or proteomics. Demographic and clinical characteristics are summarized in **Table 1**. The 6-, 12-, and 24-month overall survival and corresponding 95% confidence intervals were in the entire cohort were 74% (65–86%), 60% (49–72%), and 45% (35–58%), respectively. Median overall survival was 17.2 months (11.1–26.9 months).

**Table 1:** Patient Characteristics.

Patient Characteristics	Total
	(N = 78)
**Demographics**	
Age – yr (onset*), Mean (SD)	59.3 (11.1)
** Age Range**	28–85
** Male sex – no. (%)**	34 (44%)
** Female sex – no. (%)**	43 (56%)
** Race**	
** African – no. (%)**	4 (5%)
** Caucasian – no. (%)**	73 (94%)
** Others – no. (%)**	1 (1%)
**Histology of Primary Tumor**	
** Lung Cancer – no. (%)**	37 (47%)
** Breast Cancer – no. (%)**	16 (21%)
** Melanoma – no. (%)**	14 (18%)
** Others – no. (%)**	11 (14%)
**Overall Survival: no.**	74
** Events – no. (%)**	58 (78%)
** Followup (yr), Mean (SD)**	27.7 (31.0)
** Time of Event (yr), Mean (SD)**	17.5 (18.9)
**Distant Brain Failure-free Survival: no.**	75
** Events – no. (%)**	42 (56%)
** Followup (yr), Mean (SD)**	13.7 (20.0)
** Time of Event (yr), Mean (SD)**	10.7 (10.3)
**Disease Burden**	
** Widespread – no. (%)**	37 (47%)
** Oligometastatic – no. (%)**	35 (45%)
** None – no. (%)**	5 (6%)
** Unknown – no. (%)**	1 (1%)
**Disease Status**	
** Stable – no. (%)**	8 (10%)
** Progressive – no. (%)**	39 (50%)
** Unknown – no. (%)**	31 (40%)
**Karnofsky Performance Scale**	
** 50 – no. (%)**	1 (1%)
** 60 – no. (%)**	9 (12%)
** 70 – no. (%)**	13 (17%)
** 80 – no. (%)**	28 (36%)
** 90 – no. (%)**	26 (33%)
** Unknown – no. (%)**	1 (1%)
**Brain Metastasis at Time of Diagnosis (yr)**	
** Mean (SD)**	2.7 (3.1)
** Range**	1–23
**Type of Adjuvant Local Therapy**	
** None – no. (%)**	9 (12%)
** Gama Knife – no. (%)**	44 (56%)
** Gliasite – no. (%)**	4 (5%)
** WBRT – no. (%)**	10 (13%)
** Gliadel Wafer – no. (%)**	4 (5%)
** WBRT + Planned SRS – no. (%)**	1 (1%)
** Fractionation IMRT – no. (%)**	1 (1%)
** Unknown – no. (%)**	6 (6%)

Demographics, histology of primary tumor, survival, disease burden and disease status, patients’ performance, detection time of brain metastasis, and treatment information were comprehensively summarized in this table. WBRT, Whole Brain Radiation Therapy; SRS, stereotactic radiosurgery; IMRT, intensity modulated radiation therapy.

### Development of New Brain Metastases

The distal brain failure (DBF) free survival was 10.0 month (95% confidence interval: 7.3–27.4 month). The 6-, 12-, and 24-month freedom from distant brain failure for the entire cohort and corresponding 95% confidence intervals were 68% (57–81%), 45% (33–60%), and 37% (26–52%), respectively.

### Impact of Primary Tumor Sites on Survival

**Figure 2:**
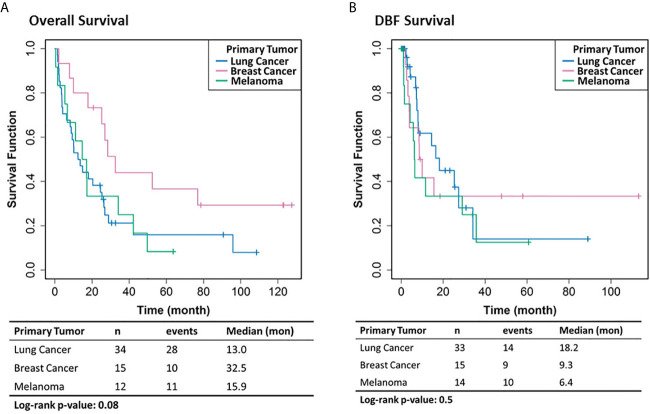
Patient survival analysis. **(A)** Overall survival. **(B)** Distant brain failure (DBF) free survival.

Patients with brain metastasis from breast cancers showed better survival than those from lung cancer or melanoma, with a median survival of 32.5 months versus 13.0 and 15.9 months, respectively (**Figure 2A**). However, the statistical significance (p-value <= 0.08) is limited due to the modest sample sizes of the lung- and skin- originated cases (n = 15 and 12, respectively). No significant difference of DBF survival was observed among patients with different primary tumor sites (**Figure 2B**).

### Patient Subtyping

**Figure 3:**
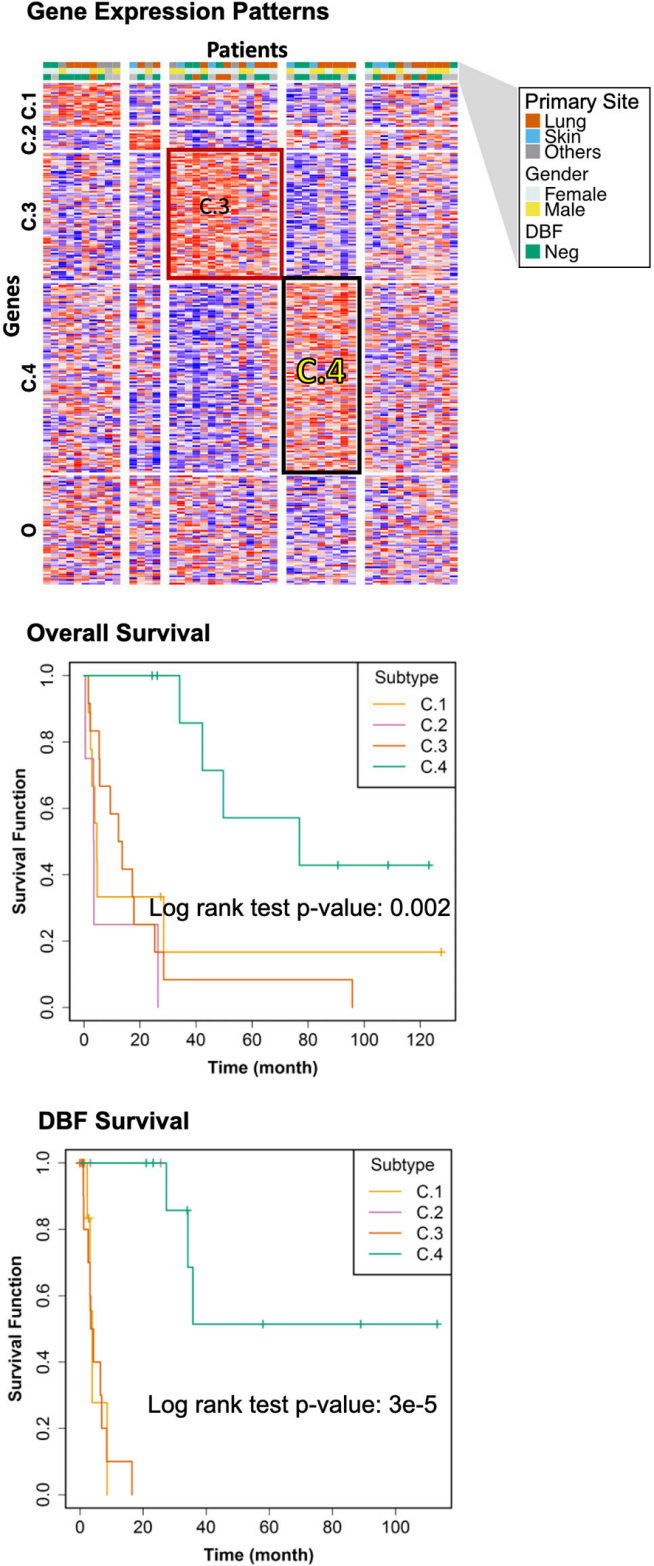
Transcriptomic Molecular Subtyping. Top: gene expression patterns across discovered patient subgroups. Columns: patients; rows: genes; color: relative gene expression; patient phenotypes were color-coded according to primary sites, genders, and events of distant brain failure (DBF). Patients in the transcriptomics cohort (n = 46) were clustered according to the gene expression. Four clusters (C.1 through C.4) were identified, with the rest patients (O) not belonging to any of these clusters. Middle: overall survival of the discovered subtypes. Bottom: DBF-free survival of the discovered subtypes.

The genomic bi-clustering analysis using sparse non-negative matrix factorization (sNMF) approach were able to characterize patients into four distinct transcriptomic molecular clusters (C.1 through C.4, **Figure 3**, top). Genes associated with each cluster were listed in **Supplementary Table 1**.

**Figure 4:**
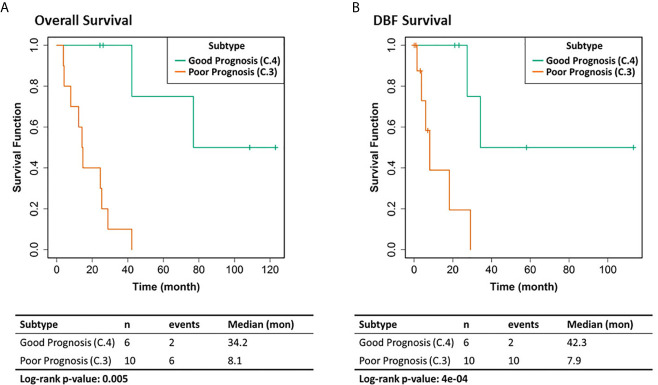
Clinical outcomes of the corresponding proteomics cohort. **(A)** Overall survival. **(B)** Distant brain failure (DBF) free survival.

We selected the C.4 cluster as the Good Prognosis Subtype, and C.3 cluster as the Poor Prognosis Subtype for further multi-omics differential analysis. The C.3 was selected for two reasons: better overlapping with proteomics and metabolomics data, and richer transcriptomic features. The survival curves of the Good Prognosis and Poor Prognosis subtypes with regards to the likelihood of death (**Figure 3**, middle) and distant brain failure after surgical resection (**Figure 3**, bottom) of a brain metastasis showed that the C.4 subtype was associated with better prognosis, while the rest three subtypes showed poor survival. Both subtypes were mapped to the proteomics cohort with patients who had both transcriptomics and proteomics data. The similar survival difference between the Good and the Poor Prognosis Subtypes in the proteomics cohort (**Figure 4**) for both overall survival (**Figure 4A**) and distant brain failure-free survival (**Figure 4B**).

### Multi-Omics Analysis

To comprehensively reveal the molecular profiles of the Good Prognosis and the Poor Prognosis subtypes, we further associated the proteomics and the metabolomics data and performed differential analysis. The enriched canonical signaling pathways were identified using the associated mRNAs and differentially expressed proteins, respectively, ranked and scored according to p-values, summarized together, and listed in **Table 2**. These pathways were further categorized into three groups: growth, immune, and migration. The mixed pathway ranking of these three categories suggested the complexity of the tumor progress.

**Table 2:** Transcriptomic and Proteomic Integrative Analysis.

Canonical Pathway	RNA-Seq	Proteomics	Score	Category
**EIF2 Signaling**	**0.509**	**5.84**	**6.349**	**Growth**	
**Regulation of eIF4 and p70S6K Signaling**	**0.209**	**5.53**	**5.739**	**Growth**	
**tRNA Charging**	**1.57**	**3.87**	**5.44**	**Growth**	
**CD28 Signaling in T Helper Cells**	**1.17**	**3.47**	**4.64**	**Immune**	
**Integrin Signaling**	**0.527**	**3.64**	**4.167**	**Migration**	
**fMLP Signaling in Neutrophils**	**0.471**	**3.56**	**4.031**	**Immune**	
**Actin Nucleation by ARP-WASP Complex**	**0.62**	**3.4**	**4.02**	**Migration**	
**Role of PKR in Interferon Induction and Antiviral Response**	**1.51**	**2.31**	**3.82**	**Immune**	
**IL-17A Signaling in Fibroblasts**	**3.57**	**0.198**	**3.768**	**Immune**	
**IL-6 Signaling**	**2.24**	**1.37**	**3.61**	**Immune**	
**Regulation of IL-2 Expr in Activated and Anergic T Lymphocytes**	**2.66**	**0.732**	**3.392**	**Immune**	
**Actin Cytoskeleton Signaling**	**0.756**	**2.58**	**3.336**	**Immune**	
**RhoGDI Signaling**	**0.26**	**3.01**	**3.27**	**Migration**	
**Acute Phase Response Signaling**	**1.18**	**2.03**	**3.21**	**Immune**	
**Ephrin Receptor Signaling**	**0.209**	**3**	**3.209**	**Migration**	
**Superpathway of Cholesterol Biosynthesis**	**2.94**	**0.198**	**3.138**	**Growth**	
**Dendritic Cell Maturation**	**0.209**	**2.86**	**3.069**	**Immune**	
**JAK/Stat Signaling**	**1.3**	**1.72**	**3.02**	**Growth**	
**PI3K Signaling in B Lymphocytes**	**2.81**	**0.198**	**3.008**	**Immune**	
**Rac Signaling**	**0.514**	**2.49**	**3.004**	**Migration**	
**PKCθ Signaling in T Lymphocytes**	**1.64**	**1.35**	**2.99**	**Immune**	
**mTOR Signaling**	**0.209**	**2.76**	**2.969**	**Growth**	
**PPAR Signaling**	**2.28**	**0.665**	**2.945**	**Growth**	
**CD27 Signaling in Lymphocytes**	**2.73**	**0.198**	**2.928**	**Immune**	
**4-1BB Signaling in T Lymphocytes**	**2.72**	**0.198**	**2.918**	**Immune**	
**Protein Ubiquitination Pathway**	**0.549**	**2.33**	**2.879**	**Growth**	
**EGF Signaling**	**0.979**	**1.88**	**2.859**	**Growth**	
**VEGF Signaling**	**0.209**	**2.65**	**2.859**	**Growth**	
**Signaling by Rho Family GTPases**	**0.403**	**2.44**	**2.843**	**Growth**	
**PDGF Signaling**	**1.19**	**1.65**	**2.84**	**Growth**	
**Selenocysteine Biosynthesis II (Archaea and Eukaryotes)**	**1.02**	**1.82**	**2.84**	**Growth**	
**Oncostatin M Signaling**	**0.36**	**2.47**	**2.83**	**Immune**	
**Cdc42 Signaling**	**0.603**	**2.16**	**2.763**	**Migration**	
**Fatty Acid β-oxidation I**	**0.209**	**2.52**	**2.729**	**Growth**	
**Estrogen-Dependent Breast Cancer Signaling**	**0.861**	**1.78**	**2.641**	**Growth**	
**Role of NFAT in Regulation of the Immune Response**	**0.71**	**1.93**	**2.64**	**Immune**	
**IL-17 Signaling**	**1.86**	**0.708**	**2.568**	**Immune**	
**Th1 Pathway**	**0.209**	**2.32**	**2.529**	**Immune**	

Signaling pathways associated with growth, immune, and migration are highlighted in orange, green, and blue colors, respectively.

Differentially abundant metabolites or metabolite-based indicators were shown in **Table 3**, demonstrating the strength of association between metabolites and the likelihood of distant brain failure and death. Significant metabolic biomarkers include glycerophospholipids such as lysophosphatidylcholines (lysoPC a C18:2, lysoPC a C20:4, lysoPC a C20:3 and lysoPC a C20:4), phosphatidylcholines (PC ae C36:0 and PC ae C44:6), amino acids (arginine, ornithine, serine, and valine), acylcarnitines (C3, C4, and C5), a sphingomyelin (SM-OH), and a biogenic amine (carnosine). Among them, half were single metabolites and the other half were metabolite-based indicators.

**Table 3:** Metabolomic Feature Analyssis.

Metabolite	Class	logFC
**lysoPC a C18:2**	**glycerophospholipids**	**1.33**
**C3**	**acylcarnitines**	**-0.70**
**Orn / Arg**	**Cust. Met. Indicator**	**0.82**
**Orn**	**aminoacids**	**0.77**
**PC ae C36:0**	**glycerophospholipids**	**-1.03**
**Arg/(Arg+Orn)**	**Cust. Met. Indicator**	**-0.62**
**Total SM-OH / Total SM-non OH**	**Cust. Met. Indicator**	**-0.94**
**lysoPC a C20:4 / lysoPC a C20:3**	**Cust. Met. Indicator**	**1.02**
**PC ae C44:6**	**glycerophospholipids**	**-0.76**
**lysoPC a C20:4**	**glycerophospholipids**	**0.83**
**Val / C5**	**Cust. Met. Indicator**	**0.54**
**Orn / Ser**	**Cust. Met. Indicator**	**0.55**
**C3 / C4**	**Cust. Met. Indicator**	**-1.71**
**Carnosine**	**biogenic amines**	**-0.89**

## Discussion

Should the multi-omics signature for distant brain failure identified in the present study be validated, it would represent a major advance in the search for brain metastasis biomarkers. First of all, patients who, based on these findings, would be biologically at high risk of distant brain failure would certainly require post-treatment surveillance imaging. Surveillance studies have suggested that patient outcomes are improved when distant brain failures are caught prior to becoming symptomatic (33). Patients at lower risk of distant brain failure could be treated more aggressively with SRS instead of WBRT. In addition, the findings from the present analysis can potentially be used to study patient primary tumors (prior to becoming metastatic) to determine if such a signature can be applied to tumors that may be at risk of ultimately developing brain metastases.

Several previous attempts have been made to discover biomarkers for brain metastasis behavior (34–37). Dohm et al. evaluated RNA-seq data from patients who underwent fine needle aspiration for newly diagnosed non-small cell lung cancer (36). These patients were subsequently followed over the natural history of their disease and the genomics of patients who developed brain metastases were compared to those who did not develop brain metastases. Two genes were identified that with an association to development of brain metastases, but a false discovery analysis was unable to confirm this association.

The CE.7 trial is a presently accruing randomized trial assessing the efficacy of WBRT vs GKRS in the population with 5–15 brain metastases (11). This trial represents an example of a modern perspective brain metastasis study in which collected serum will be submitted for genomic analysis, and then correlated to patterns of failure. Moving forward, prospective trials with strong correlative science will likely be the next step in the evolution in the elucidation of genomic biomarkers for brain metastasis behavior. Particularly because of the heterogeneity of the brain metastasis population, large trials that bank tissue or serum for genomic analysis will be crucial in such biomarker discovery.

While the genomic and proteomic signature suggest pathways involved in metastasis development, the mechanisms to explain the metabolomic associations with distant brain failure are less clear. Early data suggests that metabolomic re-programming leading to a phenotype that is more conducive for metastases is possible (38).

Lysophosphatidylcholines (lysoPCs) impact a wide range of biological and physiological functions, modulating inflammation, regulate angiogenesis, and interfere the integrity of mitochondrial membrane. Many lysophosphatidylcholines that showed differential abundance across the discovered prognostic subtypes are potential metabolomic molecular markers in cancers (39–42) as well as other chronic diseases such as diabetes and cardiovascular disease. For example, the abundance of lysoPC a C18:2, lysoPC a C20:3, lysoPC a C20:4, and PC ae C42:5 in plasma of cancer patients were found significantly different from healthy controls (39). The lysoPC a C18:2 abundance in serum is also a significant biomarker for myocardial infarction (43). The increased lysoPC a C18:2 abundance in the poor prognostic subtype may reflect specific molecular traits in the immune microenvironment responsible for the disease progression. Among potential molecular mechanisms, interleukin 6 (IL-6) signaling, dendritic cell maturation, acute phase response signaling, and CDC42 signaling enriched in the poor prognostic subtype (**Table 2**) are known to be associated with lysoPC a C18:2.

Sphingomyelins (SM) play an essential role in brain, supporting myelination of neurons and regulating brain inflammatory responses (44). Sphingomyelin hydroxylation patterns serve as promising biomarkers for a wide range of inflammation symptoms as well as the underlying signaling events such as the activity of sphingosine kinases and the associated inflammatory signaling pathways including tumor necrosis factor-alpha (TNF-α) and interleukin1-β (IL1-β) (45). It has also been previously reported that the hydroxylation patterns on sphingomyelin backbones could be leveraged to treat cancers (46). We observed significantly decreased hydroxylation of sphingomyelins (Total SM-OH/Total SM-non OH) in the discovered poor prognostic brain metastasis subtype, probably associated with the enriched inflammation pathways in this subtype.

Some tumors exhibit a hallmark metabolic pattern known as the Warburg effect, featured by the aerobic glycolysis, the increased usage of lactate, and the up-regulated activity of corresponding enzymes including lactate dehydrogenases (LDHs) and monocarboxylate transporters (MCTs) (47, 48). In our cohort, at the mRNA level, some Warburg effect genes are negatively associated with the risk of distant brain failure, with the log fold changes of MCT genes SLC16A7 and SLC6A20 as -2.89 (FDR <= 0.0047) and -3.92 (FDR <= 0.014), respectively. However, at protein level, proteins related with Warburg effect show no significant association with distant brain failure. Furthermore, the aerobic glycolysis in the discovered Poor Prognosis group is insignificant, according to the Biocrates’ AbsoluteIDQ glycolysis metabolic indicator (log fold change: 0.10, FDR: 0.90). In summary, our results suggest that the Warburg effect is not responsible for the increased distant brain failure risk in the Poor Prognosis group.

All patients recruited in this study underwent surgical resection of brain metastasis, through which tumor tissues were retrieved. Surgical resection of brain metastasis allows for acute reduction of tumor burden and mass effect, as well as improvement in cerebral edema. This often equates to improved survival, local recurrence, in up to three metastases (49, 50). Surgical resection also allows for the retrieval of tumor tissue for diagnosis and molecular characterization. Improvements in surgical technique and radiation demonstrate improvement in survival and function.

It is still far from identifying the driver mutations that can be used to stratify patients with brain metastases and improve their prognosis. Some genetic abnormalities such as RAS/PIK3CA (51) and EGFR (52, 53) mutations are common in the brain metastatic cases. However, the prognostic value of such biomarkers is understudied comparing with that in the primitive cancers. This work, one of the first in its kind, provides a comprehensive landscape of the molecular traits for the prognosis of brain metastasis patients, paves way to further understanding of such common but less studied mutations, and leads to mechanistic studies on the molecular underpins and possible targeted therapies.

There are several limitations to the present series. First of all, the study is limited in power based on the small population size. A larger population will be necessary in order to validate the findings. The study was also limited by patient selection bias given a single institution and the potential practice pattern biases that exist. We did not have access to patient primary tumors, and thus were unable to determine if the genomic signature for distant brain failure was inherent in the primary tumor, or evolved over the course of time to be present in the metastatic sample.

## Data Availability Statement

The data presented in the study are deposited in the GEO (Gene Expression Omnibus) repository, accession number GSE164150.

## Ethics Statement

The studies involving human participants were reviewed and approved by Internal Review Board of Wake Forest School of Medicine. The patients/participants provided their written informed consent to participate in this study.

## Author Contributions

JR, MS, and MC designed the experiments. JS, SQ, and MS analyzed the data. JS, JR, MS, and MC prepared the manuscript. All authors contributed to the article and approved the submitted version.

## Funding

Research reported in this publication was supported by the National Center for Advancing Translational Sciences of the National Institutes of Health under Award Number UL1TR001420. The work is partial financial support from the Indiana University Precision Health Initiative to JS.

## Conflict of Interest

The authors declare that the research was conducted in the absence of any commercial or financial relationships that could be construed as a potential conflict of interest.

## Supplementary Material

Supplementary Figure 1 The optimization of subtype number. The optimal number of clusters, also known as the optimal factorization rank in an NMF model, was determined from an overall of many statistical metrics, including the surveys of cophenetic coefficient decrease, dispersion, explained variance (evar), residues, residual sum of squares (RSS), silhouette, and sparseness.
