# Supplementary figures and images for: Multi-Omics Analysis of Brain Metastasis Outcomes Following Craniotomy
Source: Front Oncol. 2021 Apr 6;10:615472. doi: 10.3389/fonc.2020.615472 (PMC8056216; doi:10.3389/fonc.2020.615472)

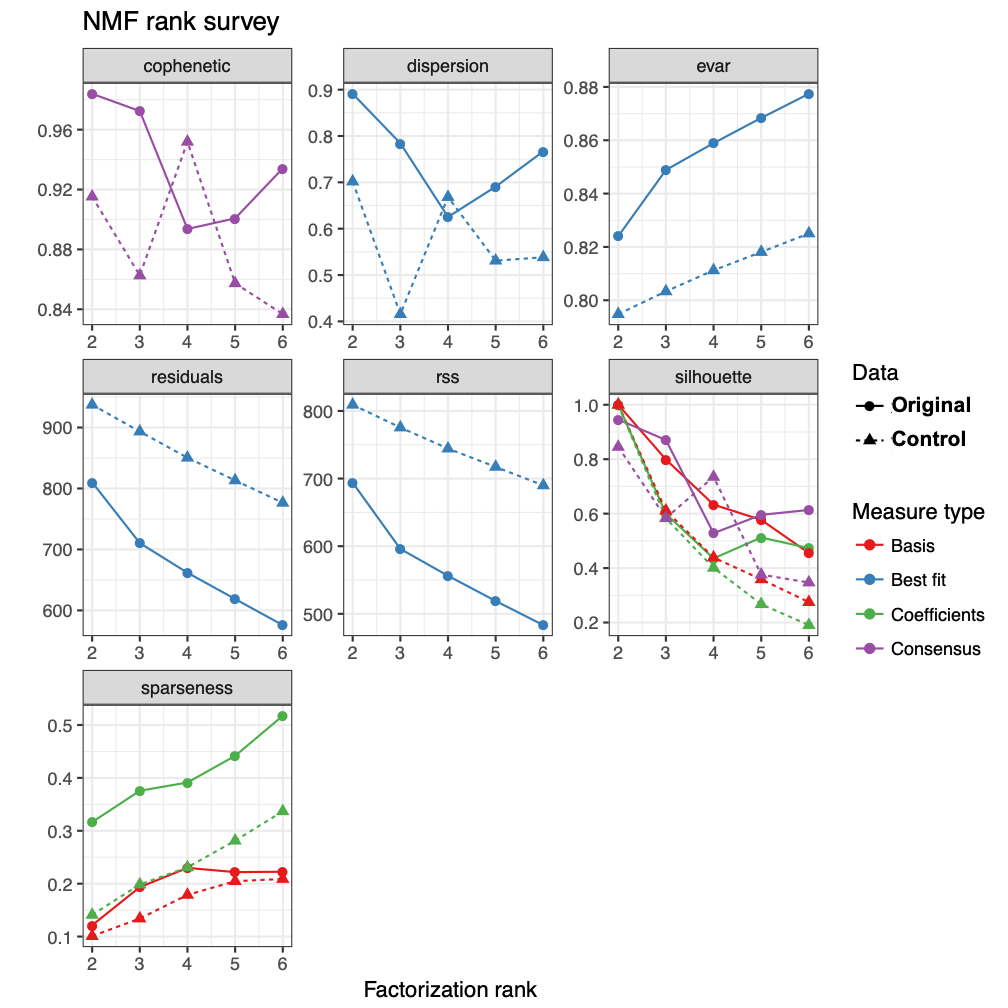

Supplement: Supplementary Figure 1 — The optimization of subtype number. The optimal number of clusters, also known as the optimal factorization rank in an NMF model, was determined from an overall of many statistical metrics, including the surveys of cophenetic coefficient decrease, dispersion, explained variance (evar), residues, residual sum of squares (RSS), silhouette, and sparseness. [file Image_1.png]
